# The role of microRNA-1246 in the regulation of B cell activation and the pathogenesis of systemic lupus erythematosus

**DOI:** 10.1186/s13148-015-0063-7

**Published:** 2015-03-14

**Authors:** Shuangyan Luo, Yu Liu, Gongping Liang, Ming Zhao, Haijing Wu, Yunsheng Liang, Xiangning Qiu, Yixin Tan, Yong Dai, Susan Yung, Tak-Mao Chan, Qianjin Lu

**Affiliations:** Department of Dermatology, Second Xiangya Hospital, Central South University, Hunan Key Laboratory of Medical Epigenomics, Changsha, Hunan 410011 China; Clinical Medical Research Center, the Second Clinical Medical College of Jinan University (Shenzhen People’s Hospital), Shenzhen, Guangdong 518020 People’s Republic of China; Division of Nephrology, Department of Medicine, University of Hong Kong, Queen Mary Hospital, Hong Kong, 999077 China

**Keywords:** Systemic lupus erythematosus, B cell, Has-miR-1246, EBF1, AKT, P53, Co-stimulatory molecules

## Abstract

**Background:**

The pathogenesis of systemic lupus erythematosus (SLE) has not yet been completely elucidated. One of the hallmarks of SLE is the production of autoantibodies by uncontrolled over-activated B cells. Early B cell factor 1 (EBF1) contributes to the development, activation, and proliferation of B cells through activation of the AKT signaling pathway. Accumulating evidence has demonstrated that several microRNAs (miRNAs) contribute to the pathogenesis of autoimmune diseases through the regulation of B cells in SLE. We aim to investigate the expression patterns of miR-1246 in B cells and its contribution to pathogenesis of SLE.

**Results:**

Our results showed that the expression of miR-1246 was significantly decreased in B cells from SLE patients. We verified that miR-1246 specifically targeted the EBF1 messenger RNA (mRNA) by interacting with its 3′-untranslated region (3′-UTR) and regulated the expression of EBF1. Transfection of miR-1246 inhibitors into healthy B cells upregulated the expression of EBF1, enhanced B cell function, and increased the production of B cell surface co-stimulatory molecules CD40, CD80, and CD86. We also observed that abnormal activation of the AKT signaling pathway was associated with decreased P53 expression, leading to the downregulation of the miR-1246 expression; and upregulation of the miR-1246 expression reversed the responsiveness of B cells by inhibiting EBF1 expression.

**Conclusions:**

Activated B cells in lupus could decrease the expression of miR-1246 through the AKT-P53 signaling pathway, which in turn enhances the expression of EBF1, thereby promoting further activation of B cells. Conversely, upregulation of miR-1246 could interrupt this amplification pathway. Our findings thus provide a theoretical framework towards the research of novel biological targets in SLE treatment.

**Electronic supplementary material:**

The online version of this article (doi:10.1186/s13148-015-0063-7) contains supplementary material, which is available to authorized users.

## Background

Systemic lupus erythematosus (SLE) is a clinically heterogeneous autoimmune disease which affects multiple organ systems and causes significant morbidity and mortality [1]. One of the hallmarks of SLE is the production of anti-nuclear autoantibodies by uncontrolled over-activated B cells [2]. The autoantibody-autoantigen immune complexes deposit in different tissues and organs, leading to chronic inflammation and tissue damage in many parts of the body. Complicated interactions between genes, environment, hormones, smoking, infections, drugs, and abnormalities in the adaptive immune system all contribute to the onset and progression of SLE [3,4]. In recent years, studies have shown that aberrant epigenetic mechanisms also play an important role in the pathogenesis of SLE [5-8].

MicroRNA (miRNA), consisting of approximately 19 to 25 nucleotides [9], is one of the principal epigenetic regulatory mechanisms which has been identified as a large, novel sub-class of endogenous noncoding RNAs (ncRNAs). MiRNAs can regulate post-transcription of protein-coding genes by guiding a protein complex known as the RNA-induced silencing complex (RISC) to bind to the 3′-untranslated region (3′-UTR) of target messenger RNAs (mRNAs) [10]. This inhibits protein translation and promotes mRNA degradation [11-13]. Current estimates suggest that through this post-transcriptional gene silencing, miRNAs can regulate at least 60% of human protein-coding genes [14]. Moreover, recent studies have shown that miRNAs plays a central role in the regulation, development, and function of the immune system and could potentially serve as disease biomarkers and therapeutic targets [9,15,16]. It has been reported that several miRNAs, including miR-155 [17], miR-146a [18], miR-326 [19], miR-23b [20], miR-126 [21], miR-142-3p/5p [22], miR-182 [23], miR-150 [24], and miR-124a [25], could modulate the pathogenesis of autoimmune diseases such as SLE, rheumatoid arthritis, and multiple sclerosis through their effects on T cells and B cell functions [26]. Nevertheless, the mechanisms by which miRNA dysregulation contributes to the pathogenesis of autoimmune diseases such as SLE have not been completely elucidated.

At present, miR-1246 has only been found in the ape and human genomes. In humans, its gene is located in chromosome two (2q31.1). Some recent studies have reported an association between aberrant miR-1246 expression and P53 regulation, thereby explaining its role in the pathogenesis of cancer and Down’s syndrome [27,28]. In response to DNA damage, p53 can induce miR-1246 expression, which then reduces the level of DYRK1A, a Down’s syndrome-associated protein kinase. In addition, a p53-miR-1246-DYRK1A-NFAT pathway has been proposed in cancer pathogenesis. It is also reported that serum miR-1246 had a strong potential to serve as a novel diagnostic and prognostic biomarker in esophageal squamous cell carcinoma [29]. However, little is known with regard to the role of miR-1246 in the pathogenesis of autoimmune diseases. Previous miRNA microarray studies by our group revealed that miR-1246 was reduced by approximately half in B cells from SLE patients compared to healthy controls.

In this study, we confirmed the expression patterns of miR-1246 by using real-time reverse transcription-polymerase chain reaction (RT-PCR) and investigated their involvement in SLE pathogenesis. We verified that miR-1246 specifically targeted early B cell factor 1 (EBF1) mRNA by interacting with its 3′-UTR. Transfection of miR-1246 inhibitors into healthy B cells could upregulate the expression of EBF1, enhance B cell function, and increase the production of B cell surface co-stimulatory molecules CD40, CD80, and CD86. On the contrary, upregulation of miR-1246 expression in over-activated B cells led to the reduction of the EBF1 level and downregulation of B cell activity. We also demonstrated that abnormal activation of the AKT signaling pathway was associated with decreased P53 expression, leading to the downregulation of the miR-1246 expression in SLE, and upregulation of the miR-1246 expression could reverse the responsiveness of B cells by inhibiting the EBF1 expression. Therefore, we concluded that active B cells in SLE patients demonstrated activation of AKT signaling leading to decreased P53 expression, which then decreased the expression of miR-1246 and enhanced the expression of EBF1 which contributed to a further activation of B cells. Increasing the level of miR-1246 could break down this pathway which led to aberrant B cell activation. These findings revealed that under-expression of miR-1246 might be an important molecular mechanism in B cell over-activation in SLE.

## Results

### Decreased miR-1246 expression in SLE B cells

**Figure 1:**
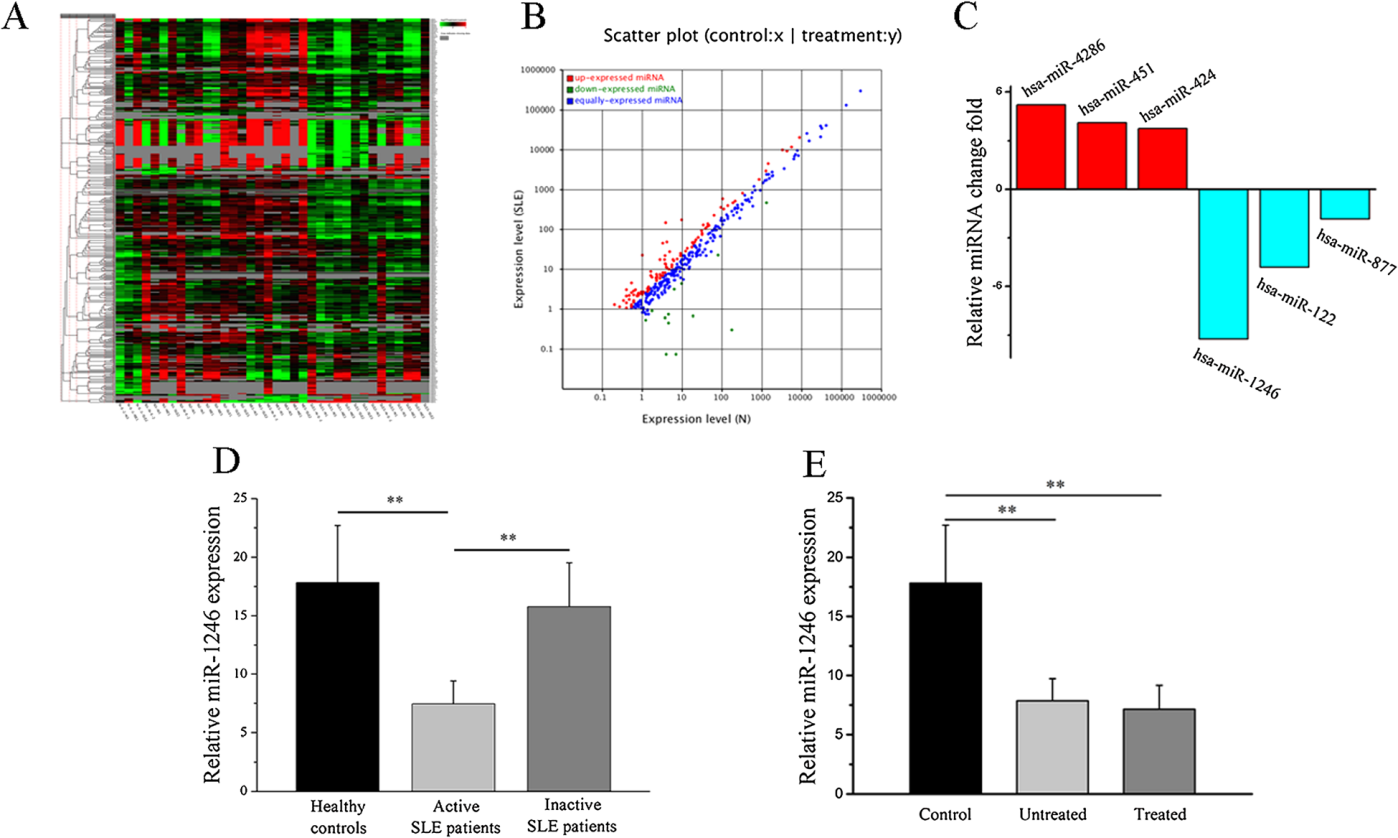
**Decreased has-miR-1246 expression in B cells from systemic lupus erythematosus (SLE). (A)** High-throughput miRNA microarray of the activities of 371 miRNAs isolated from B cells of healthy controls (*n* = 11) and active SLE patients (*n* = 11). Imbalance of green and red signal signifies non-equivalent activities. **(B)** Scatter plot of the results in (A); down-expressed miRNAs in active SLE patient samples are indicated by the green oval. **(C)** Fold-change of the six miRNAs was found to differ between SLE and control samples. **(D**, **E)** Expression of miR-1246 as measured by miRNA real-time reverse transcription-polymerase chain reaction (RT-PCR) in B cells from 30 active SLE patients (19 were being treated with corticosteroids, antimalarials, or immunosuppressive agents and 11 were untreated), 20 inactive SLE patients, and 20 age- and sex-matched healthy control subjects. Transcript levels were significantly reduced in patients with active SLE, regardless of whether they were receiving concurrent medications, while no significant difference between healthy controls and inactive SLE patients. Bars in (D, E) show the mean ± SD results in 20 healthy donors, 30 patients with active SLE, and 20 patients with inactive SLE. All experiments were performed in triplicate. (**P < 0.01).

In a previous study, our group conducted a high-throughput miRNA microarray of the activities of 371 miRNAs isolated from B cells of healthy controls (*n* = 11) and active SLE patients (*n* = 11). Among the 371 miRNAs, we observed that the expression level of miR-1246 was reduced by approximately half in B cells from active SLE patients compared with healthy controls (Figure 1A, B, C). Growing evidence has indicated that aberrant miRNA expression in B cells plays an important role in SLE pathogenesis [16]. In this study, we confirmed the microarray results through real-time RT-PCR analysis using samples from an additional 30 active SLE patients, 20 inactive patients, and 20 healthy controls.

We found that miR-1246 was significantly downregulated in B cells from active SLE patients (Figure 1D). We also compared the expression of miR-1246 in B cells between 20 inactive SLE patients and 20 healthy controls and observed no significant difference (Figure 1D), so we took the active SLE patients as our objectives. Among the 30 active SLE patients, 19 were being treated with corticosteroids, antimalarials, or immunosuppressive agents (Additional file 1: Table S1). To determine whether drug treatment accounted for the altered miRNA expression patterns observed in SLE samples, we compared the miR-1246 expression in untreated SLE patients and treated patients. There was no significant difference in the expression of miR-1246 between the two groups (Figure 1E). In addition, we treated the active SLE patients’ B cells with prednisone *in vitro* and found no effect on the miR-1246 expression (data not shown). Furthermore, we did not observe any correlation between miR-1246 levels and disease activity of active SLE patients as assessed by the Systemic Lupus Erythematosus Disease Activity Index (SLEDAI) (data not shown).

### Identification of miR-1246 targeting mRNAs in SLE B cells

**Figure 2:**
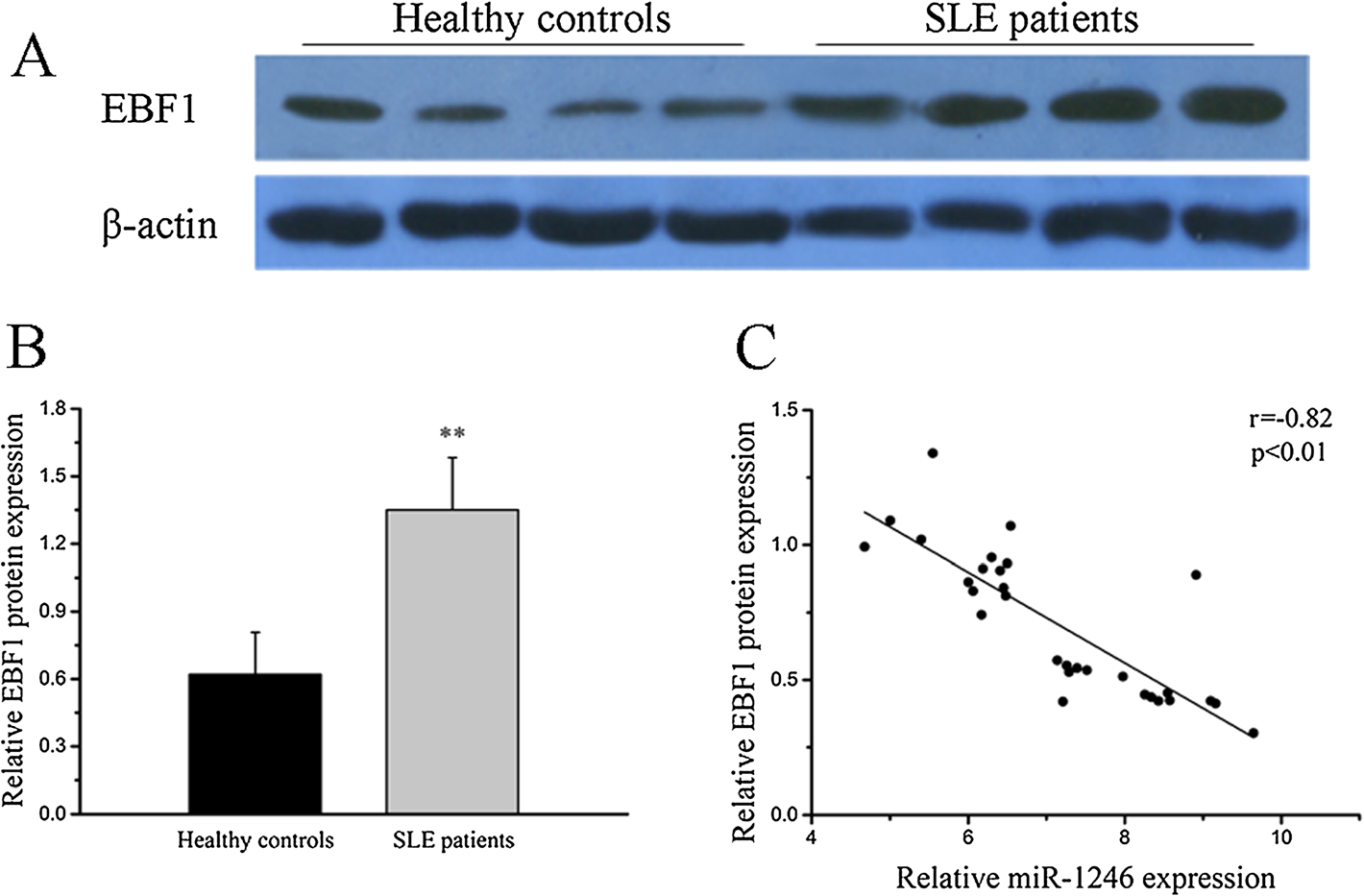
**Identification of miR-1246 target mRNAs in systemic lupus erythematosus (SLE) B cells. (A**, **B)** Early B cell factor 1 (EBF1) protein level in B cells from active SLE patients (*n* = 30) and healthy controls (*n* = 20). EBF1 expression was significantly increased in active SLE B cells compared with that in control B cells. Representative Western blotting and quantitative analysis of the band intensities of control (*n* = 4) and SLE (*n* = 4) samples normalized to *β*-actin is shown at the bottom. **(C)** In B cells derived from active patients with SLE, has-miR-1246 transcript levels were negatively correlated with the levels of EBF1 protein (*r* = −0.82, *P* < 0.01, *n* = 30). (**P < 0.01).

**Figure 3:**
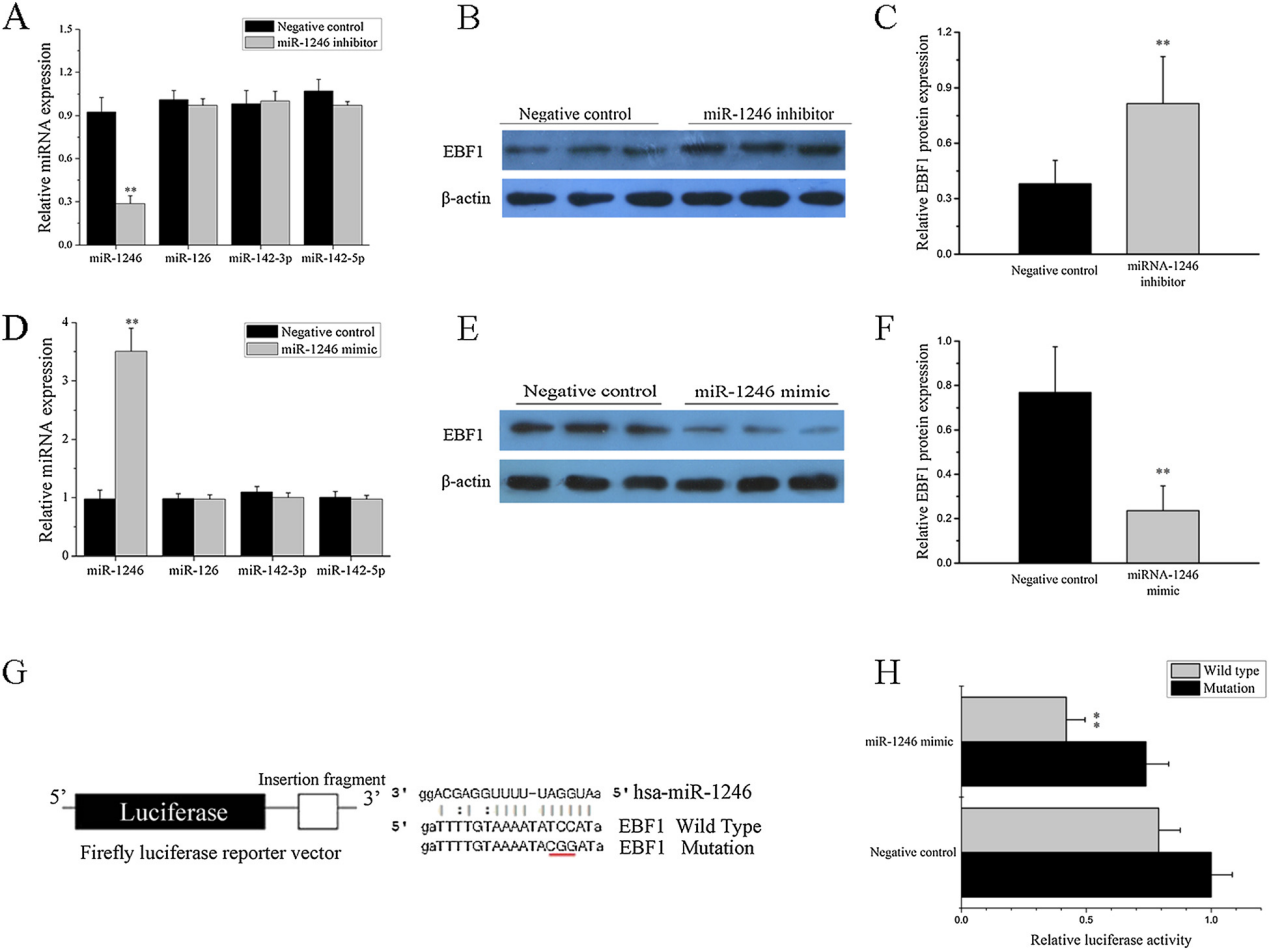
**Verification of miR-1246 target genes. (A**-**C)** The expression levels of miR-1246, miR-126, miR-142-3p, and miR-142-5p (A) and early B cell factor 1 (EBF1) protein (B, C) were analyzed after transfection with miR-1246 inhibitor or inhibitor control. **(D**-**F)** The expression levels of miR-1246, miR-126, miR-142-3p, miR-142-5p (D), and EBF1 protein (E, F) were analyzed after transfection with the miR-1246 mimic or mimic control. Bars show the mean ± SD results in three healthy donors or three patients with active SLE. All experiments were performed in triplicate. The Western blot image is a representative image (*n* = 3). **(G)** Schematic representation of the *EBF1* luciferase reporter construct is shown. The sequence of the miR-1246 binding site in the 3′-untranslated region (3′-UTR) of *EBF1* (gray box) is shown on the left. Mutated residues are shown in red. **(H)** Relative firefly luciferase activity in Jurkat cells co-transfected with an empty vector (mimic control) or an miR-1246 mimic, together with luciferase reporter constructs containing either a wild-type (WT) or a mutated (Mut) *EBF1* 3′-UTR are shown. Values in (H) are the mean ± SD results from three independent experiments. (**P < 0.01).

According to the TargetScan and miRBase bioinformatic software, EBF1, which is required for the proliferation, survival, and signaling of pro-B cells and peripheral B cell subsets, including B1 cells and marginal zone B cells [30], is a predicted target of miR-1246. To better understand the relationship between miR-1246 and EBF1, we plotted miR-1246 expression levels (measured by real-time RT-PCR) from individual SLE B cell lysates (*n* = 30) against EBF1 protein levels (measured by Western blotting) from the same samples (Figure 2A, B). In this case, a strong inverse correlation was observed (Figure 2C). To explore whether miR-1246 directly regulates EBF1, we transfected primary B cells from three healthy donors with a miR-1246 inhibitor or a control miRNA. Two days after transfection, a 3.45-fold reduction of miR-1246 level was observed while levels of the unrelated miR-126, miR-142-3p, and miR-142-5p remained unchanged (Figure 3A), and the level of EBF1 protein was significantly increased compared with negative controls (Figure 3B, C). Consistent with this finding, transfection of a miR-1246 mimic into SLE B cells induced a 3.72-fold upregulation of miR-1246 expression while those of the unrelated miR-126, miR-142-3p, and miR-142-5p remained unchanged (Figure 3D) and a significantly decreased level of EBF1 protein (Figure 3E, F).

To determine whether EBF1 is a direct target of miR-1246, a luciferase assay was performed. We constructed plasmids containing the firefly luciferase reporter ORF fused downstream to a segment of the EBF1 3′-UTR containing the wild-type putative miR-1246 binding sequence (EBF1WT-luciferase) or the equivalent segment containing three-point mutations in the miR-1246 binding sequence (EBF1Mut-luciferase) (Figure 3G). The constructs were then co-transfected into Jurkat cells together with the miR-1246 mimic or mimic control, and luciferase activity was measured 48 h after transfection. EBF1WT-luciferase activity, but not EBF1Mut-luciferase activity, was significantly reduced in cells co-transfected with the miR-1246 mimic (Figure 3H). The results showed that miR-1246 significantly reduced EBF1WT-luciferase activity. Collectively, these data provide evidence that downregulation of miR-1246 leads to increased EBF1 protein levels in SLE B cells.

### MiR-1246 represses B cell responsiveness

**Figure 4:**
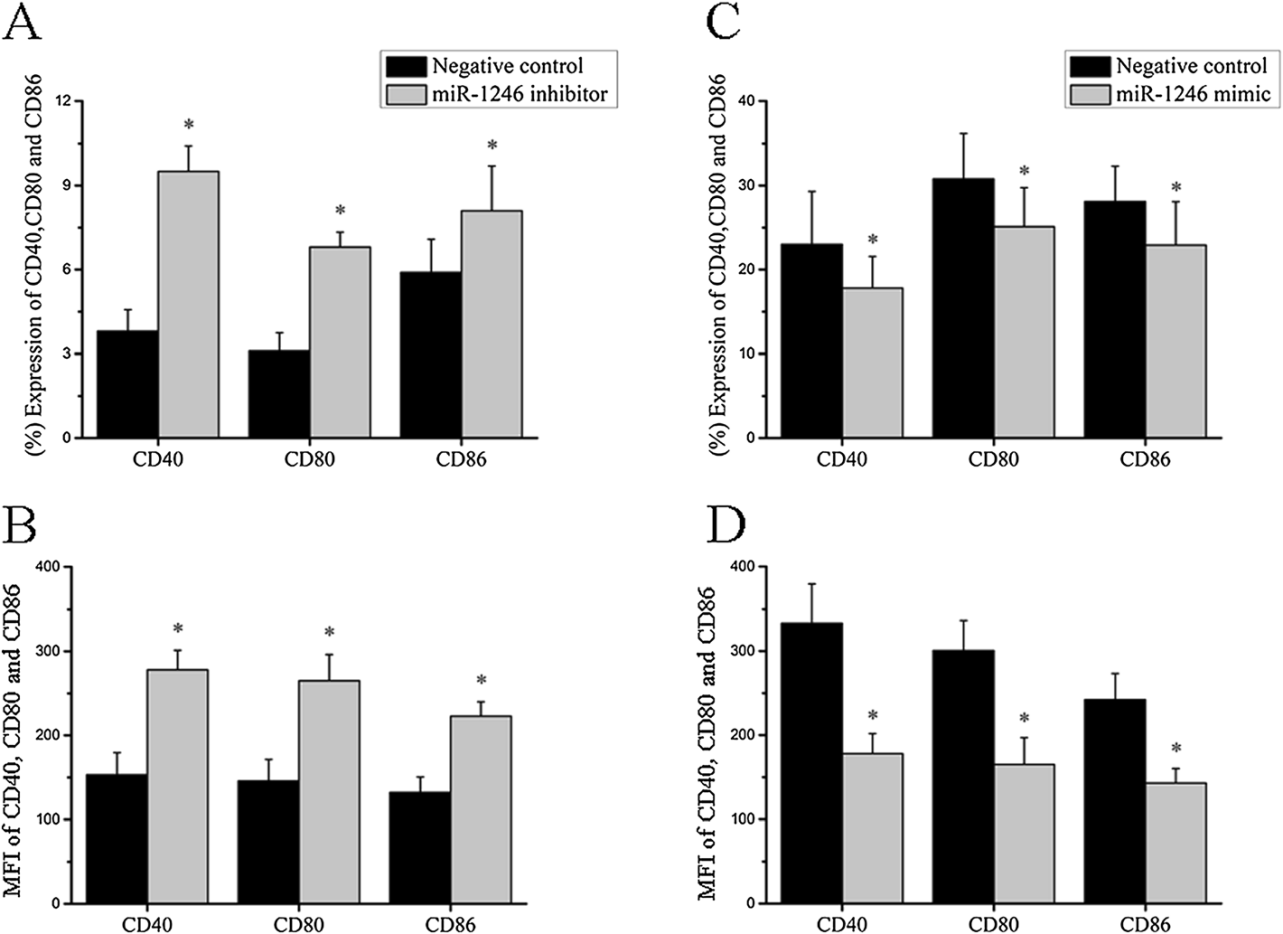
**MiR-1246 represses B cell responsiveness. (A**, **B)** Inhibiting miR-1246 expression in healthy controls’ B cells increases CD40, CD80, and CD86 expression and enhances B cell responsiveness. All panels, normal B cells were transfected with miR-1246 inhibitor or inhibitor control. Transfected cells were stained with PE-Cy7-conjugated anti-human CD40, FITC-conjugated anti-human CD80, PerCP-Cy5.5-conjugated anti-human CD86, and APC-conjugated anti-human CD19 and analyzed by flow cytometry, percentage (A), and mean fluorescence intensity (MFI) (B) for CD40, CD80, and CD86 in normal B cells inhibiting miR-1246 and in inhibitor controls (*P < 0.05). **(C**, **D)** Overexpression of miR-1246 in SLE B cells decreases CD40, CD80, and CD86 expression and reduces B cell responsiveness. All panels, SLE B cells were transfected with miR-1246 mimic or mimic control. Transfected cells were stained with PE-Cy7-conjugated anti-human CD40, FITC-conjugated anti-human CD80, PerCP-Cy5.5-conjugated anti-human CD86, and APC-conjugated anti-human CD19 and analyzed by flow cytometry, percentage (C), and MFI (D) for CD40, CD80, and CD86 in SLE B cells overexpressing miR-1246 and in mimic controls (*P < 0.05). All data represent the mean ± SD results of three independent experiments. All experiments were performed in triplicate.

To determine whether miR-1246 downregulation is sufficient to induce responsiveness of B cells *in vitro*, we transfected a miR-1246 inhibitor or an inhibitor control into B cells isolated from three healthy donors. Cells were collected after 48 h of treatment. Flow cytometric analysis was then performed to determine the levels of CD40, CD80, and CD86 expressed in B cells. Compared to control-transfected B cells, we observed significantly increased levels of CD40, CD80, and CD86 in miR-1246-deficient cells (Figure 4A, B). To explore whether miR-1246 downregulation is necessary for autoimmune reactivity in patients with SLE, we transfected primary B cells from active SLE patients with a miR-1246 mimic or a control miRNA. The results showed significantly decreased levels of CD40, CD80, and CD86 in SLE B cells transfected with the miR-1246 mimic, as compared to control-transfected cells (Figure 4C, D). The actual flow cytometry profiles are provided in supplementary materials (Additional file 2: Figure S1 and S2). Taken together, these results suggest that downregulated miR-1246 expression in healthy B cells can increase B cell function and promote B cell hyperresponsiveness, which means miR-1246 is a negative regulator of B cell activation, and that induction of over-expression of miR-1246 in SLE B cells may be a means to reverse B cell hyperactivity.

### Relationship between B cell activity and miR-1246

**Figure 5:**
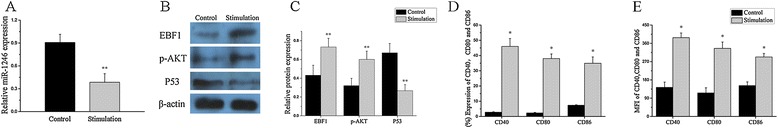
**The relationship between B cell activity and miR-1246. (A)** Measurement of miR-1246 level after stimulated with anti-IgM and anti-CD40 antibodies or PBS (control) in normal B cells. **(B**, **C)** Representative Western blotting results and densitometric analysis for early B cell factor 1 (EBF1) protein level, AKT phosphorylation, and P53 protein level in normal B cells after stimulated with anti-IgM and anti-CD40 antibodies and PBS (control). **(D**, **E)** Percentage (D) and mean fluorescence intensity (MFI) (E) of CD40, CD80, and CD86 in normal B cells stimulated with anti-IgM, anti-CD40, and PBS (control). Data are presented as the mean ± SD of the same experiments performed in three healthy donors. (*P < 0.05; **P < 0.01).

**Figure 6:**
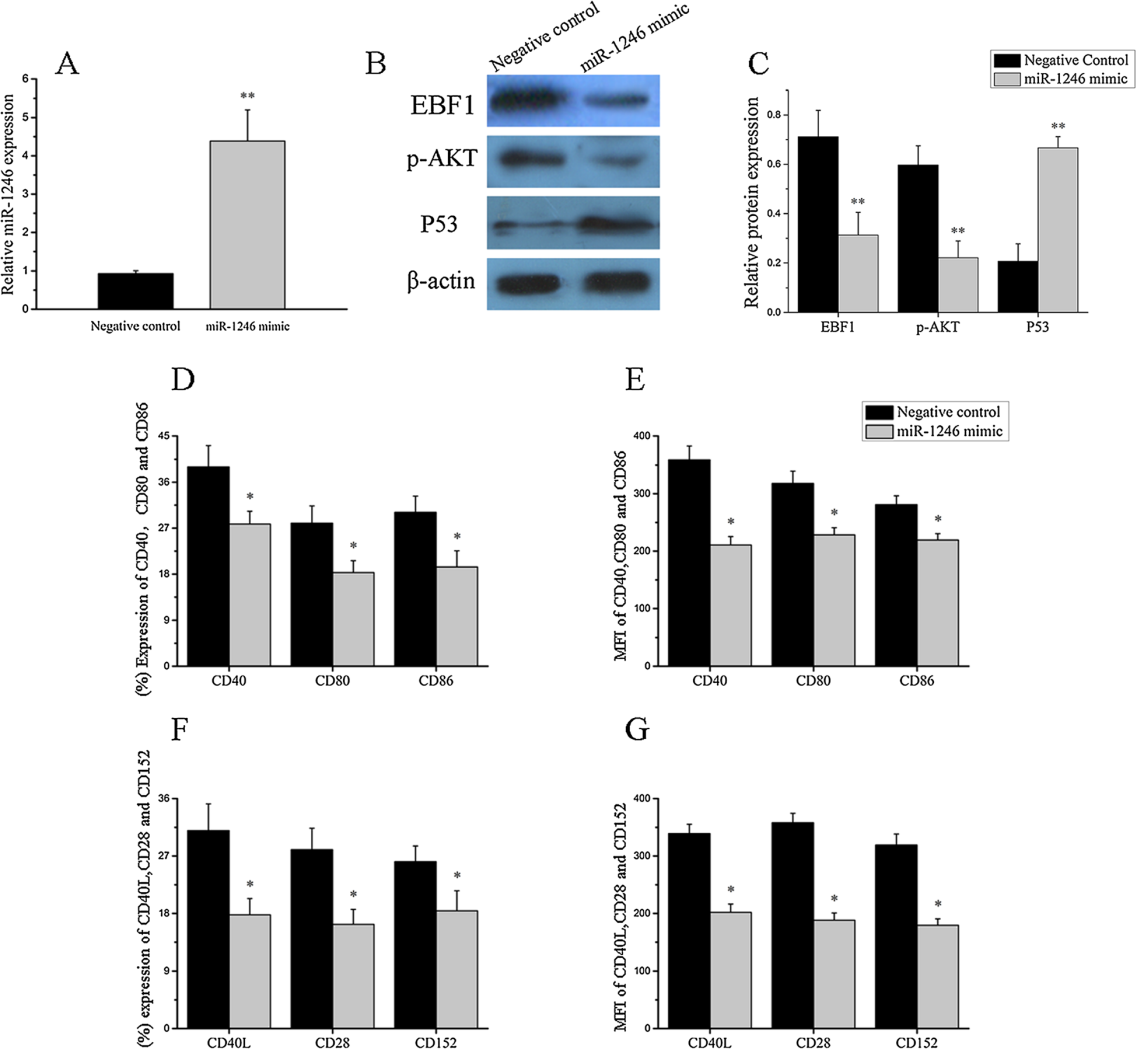
**The relationship between B cell activity and miR-1246. (A**-**E)** MiR-1246 expression (A), early B cell factor 1 (EBF1) protein level, AKT phosphorylation and P53 protein level (B, C), percentage (D), and mean fluorescence intensity (MFI) (E) of CD40, CD80, and CD86 in stimulated B cell transfected with miR-1246 mimic and mimic control. CD4^+^T cells which were isolated from the same healthy control were cultured in RPMI 1640 medium with 10% FBS, 100 U/ml penicillin G, and streptomycin. After stimulation and transfection, B cells were co-cultured with autologous CD4^+^T cells at a ratio of 4:1 in 96-well round-bottomed plates. At 24 h, percentage **(F)** and MFI **(G)** of CD40L, CD28, and CD152 were measured by flow cytometry. The Western blot image is a representative image. Data are presented as the mean ± SD of the same experiments performed in three healthy donors. (*P < 0.05; **P < 0.01).

Since B lymphocyte activation can affect the expression of miRNAs, we explored whether miR-1246 downregulation in SLE patients is a consequence of B cell hyperactivity by stimulating healthy control B cells with anti-IgM in the presence of anti-CD40 or PBS (blank control), then measured miR-1246 expression by real-time RT-PCR; EBF1 protein level, AKT phosphorylation, and p53 protein levels by Western blot; and CD40, CD80, and CD86 protein levels by flow cytometry. Next, we transfected a miR-1246 mimic or control miRNA into stimulated B cells from healthy controls, then tested miR-1246 expression by real-time RT-PCR; EBF1 protein level, AKT phosphorylation, and p53 protein level by Western blot; and CD40, CD80, and CD86 protein levels by flow cytometry. Furthermore, after stimulation and transfection, B cells were co-cultured with autologous CD4^+^T cells at a ratio of 4:1 in 96-well round-bottomed plates. After 24 h, CD40L, CD28, and CD152 protein levels on CD4^+^T cells were measured by flow cytometry. The results showed significant downregulation of the miR-1246 expression and p53 protein level and upregulation of the EBF1 expression and AKT phosphorylation level in stimulated B cells from healthy control (Figure 5A, B, C). CD40, CD80, and CD86 expression levels were increased in response to the stimulation (Figure 5D, E). The actual flow cytometry profiles are provided in supplementary materials (Additional file 2: Figure S3). In contrast, after miR-1246 mimic transfection, expression of miR-1246 and P53 protein were significantly increased, while EBF1 protein expression and AKT phosphorylation level were sharply decreased (Figure 6A, B, C), and CD40, CD80, and CD86 expression were greatly decreased (Figure 6D, E). The actual flow cytometry profiles are provided in supplementary materials (Additional file 2: Figure S4). After co-culture of miR-1246-mimic-transfected stimulated B cells with CD4^+^T cells, expression of CD40L, CD28, and CD152 were significantly decreased (Figure 6F, G). The actual flow cytometry profiles are provided in supplementary materials (Additional file 2: Figure S5).

**Figure 7:**
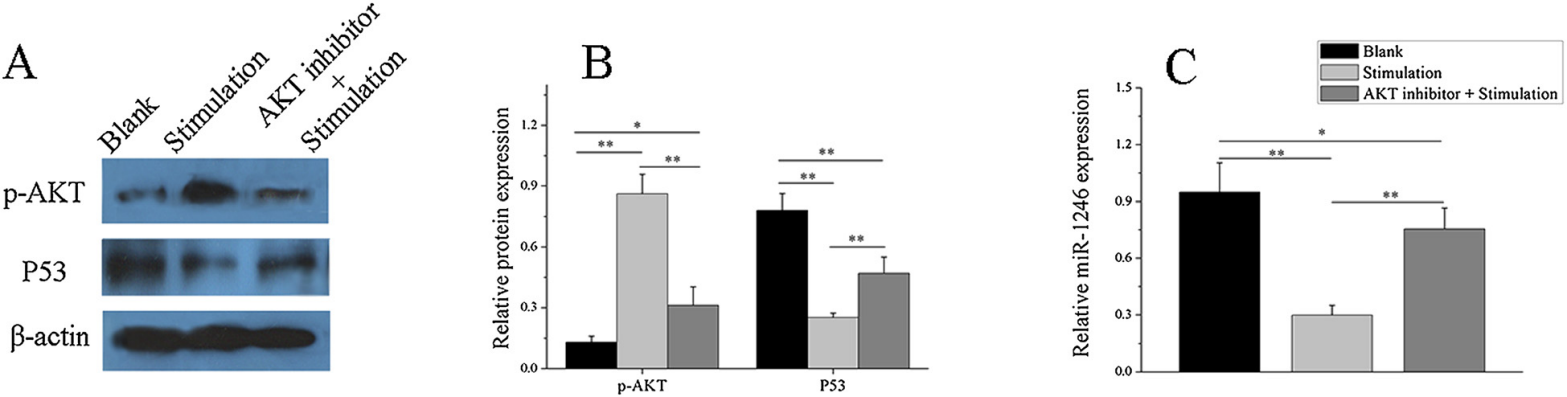
**The relationship between B cell activity and miR-1246.** We treated three healthy B cells with PBS, anti-IgM and anti-CD40 antibodies, or AKT inhibitor pre-treated + anti-IgM and anti-CD40 antibodies. **(A**-**C)** Increased AKT protein phosphorylation and decreased P53 protein and miR-1246 expression in anti-IgM and anti-CD40 antibodies-treated healthy B cells, compared with PBS-treated healthy B cells. Decreased AKT protein phosphorylation and increased P53 protein and miR-1246 expression in pre-treated AKT inhibitor + anti-IgM and anti-CD40 antibodies-treated healthy B cells compared with anti-IgM and anti-CD40 antibodies-treated healthy B cells. The Western blot image is a representative image. Data are presented as the mean ± SD of the same experiments performed in three healthy donors. (*P < 0.05; **P < 0.01).

Furthermore, we also investigated the importance of AKT signaling in the regulation of miR-1246 and P53 expression. We treated normal healthy B cells with anti-IgM and anti-CD40 antibodies or AKT inhibitor pre-treated + anti-IgM and anti-CD40 antibodies, respectively. Finally, we observed significantly increased AKT protein phosphorylation and decreased P53 protein and miR-1246 expression in anti-IgM and anti-CD40 antibodies-treated healthy B cells compared with PBS-treated healthy B cells (blank control). While we observed significantly decreased AKT protein phosphorylation, we also observed increased P53 protein and miR-1246 expression in pre-treated AKT inhibitor + anti-IgM and anti-CD40 antibodies-treated healthy B cells compared with anti-IgM and anti-CD40 antibodies-treated healthy B cells (Figure 7A, B, C). Take all together, these findings indicate that activated B cell receptor (BCR) signaling may contribute to B cell AKT signaling activation and inhibition of P53 expression, which downregulates miR-1246 expression, leading to the overexpression of EBF1 that further activates B cells. When we upregulate the miR-1246 expression, this positive feedback loop can be broken thereby inhibiting further B cell activation. Thus, we concluded that downregulation of miR-1246 in B cells is not just a consequence of increased lymphocyte activity, but also a potential cause of SLE autoimmunity.

### Regulation of miR-1246 expression by AKT-P53 signaling pathway in B cells from SLE patients

**Figure 8:**
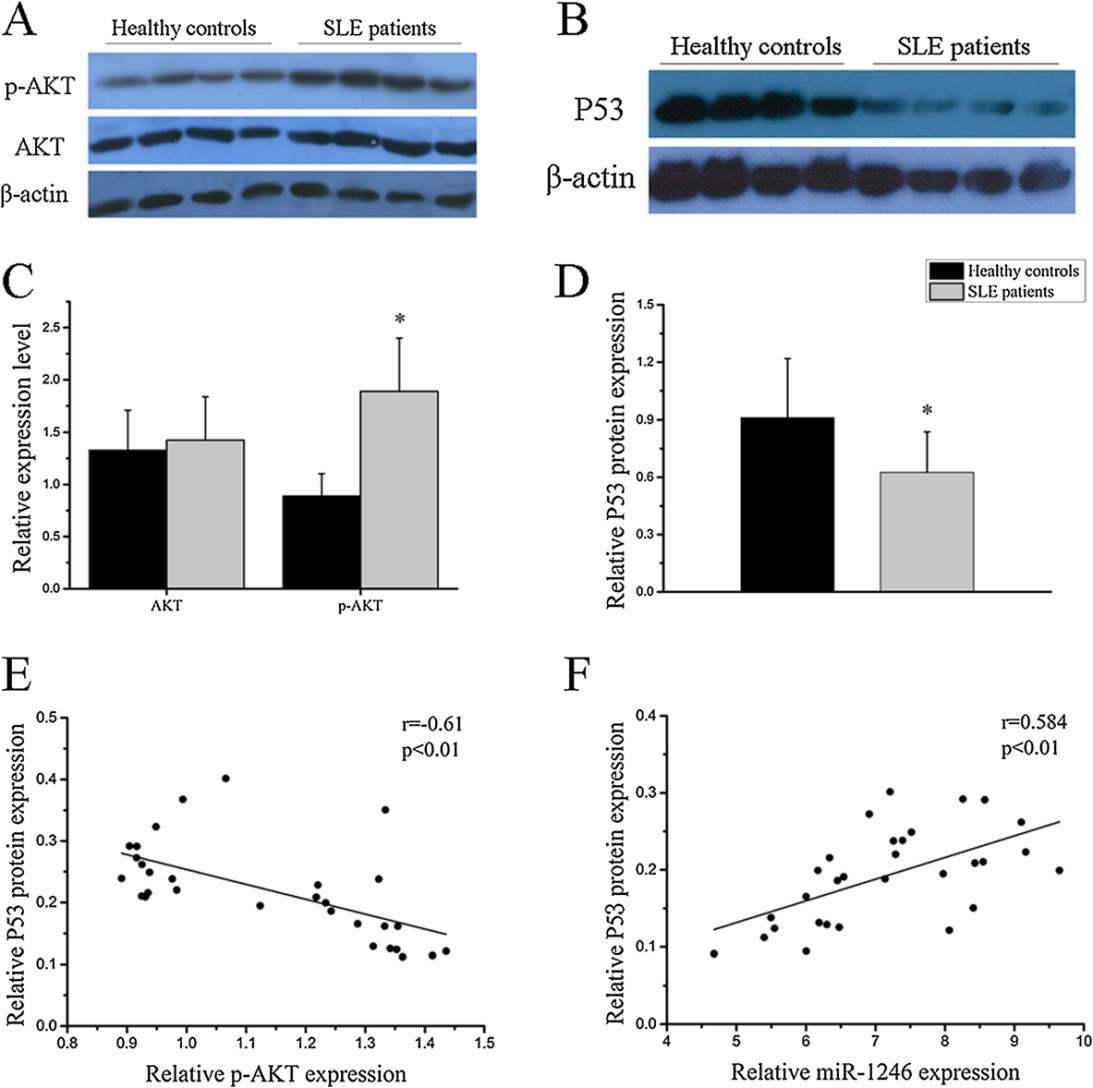
**The regulation of miR-1246 expression by AKT-P53 signaling pathway in B cells from systemic lupus erythematosus (SLE) patients. (A**, **B)** AKT phosphorylation was markedly increased, but no difference in AKT protein expression **(A**, **C)**, while P53 protein level was sharply downregulated **(B**, **D)** in B cells from active SLE patients compared to healthy controls. AKT phosphorylation levels were negatively correlated with P53 protein expression levels **(E)** while P53 protein levels were positively correlated with miR-1246 expression levels in B cells from active SLE patients **(F)**. The Western blot images are representative images (*n* = 4). Bars in (C) and (D) show the mean ± SD results in 20 healthy donors and 30 active patients with SLE. (*P < 0.05).

**Figure 9:**
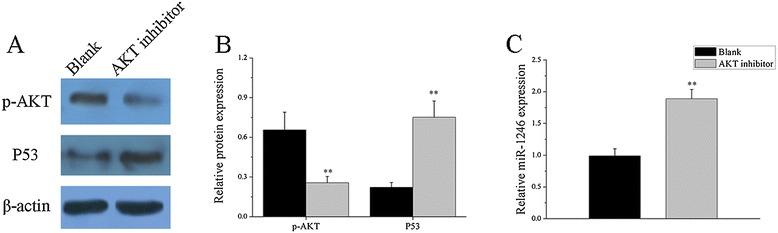
**The regulation of miR-1246 expression by AKT-P53 signaling pathway in B cells from SLE patients.** AKT protein phosphorylation, P53 protein levels, and miR-1246 expression were detected in AKT inhibitor-treated SLE B cells compared with PBS-treated SLE B cells. **(A**-**C)** AKT phosphorylation was markedly decreased, while P53 protein level and miR-1246 expression were sharply upregulated in AKT inhibitor-treated SLE B cells compared with PBS-treated SLE B cells. The Western blot image is a representative image. Data are presented as the mean ± SD of the same experiments performed in three active SLE patients. (**P < 0.01).

In previous studies, we found that miR-1246 was significantly downregulated in B cells from SLE patients in comparison with healthy controls. And we also demonstrated that stimulation of the BCR signaling pathway increased AKT phosphorylation level and decreased p53 protein and miR-1246 expression. These data indicate that the AKT-P53 signaling pathway might be the upstream factor leading to altered miR-1246 expression. Therefore, in this part, we aim to confirm the dysregulation of the AKT-P53 signaling pathway and whether it contributes to the downregulation of miR-1246 expression in SLE B cells. We found that AKT phosphorylation was markedly increased with no obvious difference in AKT protein level (Figure 8A,C), while P53 protein level was markedly downregulated (Figure 8B, D) in B cells from active SLE patients compared to healthy controls. AKT phosphorylation levels were negatively correlated with p53 protein expression levels (Figure 8E), while p53 protein levels were positively correlated with miR-1246 expression levels in B cells from SLE patients (Figure 8F). Furthermore, we also investigated whether inhibition of AKT signaling could affect miR-1246 and P53 expression in B cells from active SLE patients. We treated active SLE B cells with AKT inhibitor, then we observed significantly decreased AKT protein phosphorylation and increased P53 protein and miR-1246 expression in AKT inhibitor-treated SLE B cells compared with the blank (PBS-treated) (Figure 9A, B, C). Thus, we conclude that reduced miR-1246 levels in B cells of SLE patients might be due to inhibition of p53 protein expression by AKT phosphorylation.

## Discussion

SLE is a potentially lethal systemic autoimmune disease with multi-organ damage. The intricate clinical manifestations of SLE could immunologically be attributed to the loss of self-tolerance and the production of autoantibodies by B cells [31]. The dysregulated expression profile of microRNAs plays an important role in the pathogenesis of SLE [8]. In the last decades, several independent profiling studies on immune-cell-derived or circulating miRNA expression reported significant differences between SLE patients and healthy controls, which provides useful information for understanding SLE pathogenesis and the development of biomarkers for diagnosis, prognosis, and novel therapeutic targets [16]. And the impact of specific miRNA-mediated regulation on function of major immune cells, such as T and B lymphocytes, in lupus is also discussed. For example, defective miR-7 regulation of PTEN, which regulates normal signaling through the BCR, contributes to B cell hyperresponsiveness in SLE [32]. In this study, we screened the expression profile of microRNAs in B cells from active SLE patients and healthy controls through microarray. We observed and validated a significant decrease of miR-1246 expression in B cells only from active SLE patients, but not in inactive SLE patients or healthy controls, which implicated a role of miR-1246 in active SLE and might be developed into a potential biomarker and a novel therapeutic target of active SLE.

Previous studies found that the expression of miR-1246 increased significantly in some solid cancers, such as colon cancer, hepatocellular carcinoma, pancreatic cancer, and esophageal squamous cell carcinoma, and might be used as diagnostic and prognostic biomarkers in these diseases [29,33-35]. The upregulation of miR-1246 could downregulate the expression levels of its target genes to mediate the proliferation, invasion, and migration of cancer cells [34-36]. However, the role of miR-1246 in autoimmune diseases has been poorly studied. Our results demonstrated that the downregulation of miR-1246 in active B cells from SLE patients could in turn enhance the responsiveness of B cells through the upregulation of its target gene EBF1, which has been identified by luciferase reporter assay.

EBF1 is a transcriptional factor that has a central role in the development of B cells. EBF1 persists as homodimers in solution and when it binds to sequences of promoters that loosely fit the consensus 5′-CCCNNGGG-3′ [37]. It is a regulator of B cell lineage specification and commitment and is expressed throughout B cell development up to the plasma cell stage [38]. In the absence of EBF1, progenitor cells fail to express classical markers of B cells, including immunoglobulins [39]. EBF1 was found to be necessary for B cell maturation, and upregulation of EBF1 in B cells contributes to autoreactivity by activating B cell functions [40,41]. However, there are few reports about EBF1 involved in the pathogenesis of SLE. Interactions between T and B cells depend on engagement of appropriate B cell co-stimulatory molecules [42]. Among the most important co-stimulatory molecules are those including CD40 and CD80/86 which are expressed on antigen-presenting cells (DC, macrophages, and B cells) [43-45]. CD80, CD86, and CD40 play key roles in the polyclonal B cell activation [46] upon interaction with CD28, CD152, and CD40L and, in particularly, enhance IgG secretion [42,47,48]. In our studies, we found that elevation of the expression level of miR-1246 in lupus B cells could decrease the expression of EBF1 causing the downregulation of CD40, CD80, and CD86 which are co-stimulatory molecules required for full B cell activation and IgG secretion [47,49]. On the contrary, a decrease of the level of miR-1246 in normal B cells would lead to the upregulation of EBF1 and activation of B cells, which means that miR-1246 acts as a negative regulator of B cell activation and a protective factor against SLE. CD40 belongs to the tumor-necrosis-factor super family. On activation upon ligation with CD40L expressed on activated T cells, CD40 provides a co-stimulatory signal that induces B cell proliferation and differentiation with subsequent antibody production [50]. CD40 also promotes CD80/86 expression and provides further stimulation signals to T cells [51,16]. CD80/86 belong to the immunoglobulin (Ig) superfamily (IgSF). Interaction between CD80/86-CD28/CD152 is crucial for T cell activation [46]. Consequently, the stimulated T cells further signal the B cells to proliferate and differentiate into antibody-producing plasma cells and CD80/CD86 signaling directly regulates IgG secretion by previously activated B cells [52].

Activation of phosphoinositide 3-kinase (PI3K) has been demonstrated to play a pivotal role in B cell proliferation and survival. It initiates a signaling pathway involving a serine-threonine kinase AKT which is essential for B cell functions and decisions. However, the AKT signaling pathway has complex roles in B cell fate and the exact mechanisms remain to be elucidated [53,54]. In this study, we stimulated normal B cells with anti-IgM and anti-CD40, and we observed that the expression level of miR-1246 decreased, whereas EBF1 expression level increased. In addition, the B cells were activated and CD40, CD80, and CD86 expression levels were upregulated. Since miR-1246 has been proved to be a target microRNA of transcriptional factor P53 [27,28] and P53 can be mediated by AKT signaling pathway which plays an important role in cellular proliferation and growth signaling [55,56], we examined the levels of miR-1246, pAKT, and the proteins of EBF1, P53, CD40, CD80, and CD86 in activated normal B cells. The results showed that the protein levels of EBF1, CD40, CD80, and CD86 and the phosphorylation level of AKT increased and expression of miR-1246 and the protein level of P53 decreased. Taken all together, we hypothesized that activation of B cells could cause the phosphorylation of AKT which inhibited P53 expression leading to the downregulation of miR-1246 transcription and the upregulation of EBF1 which in turn promotes further activation of B cells. Moreover, when we transfected activated normal B cells with miR-1246 mimics, we found that the protein level of EBF1 and the phosphorylation level of AKT decreased while the protein level of P53 increased which led to the further elevated expressional level of miR-1246, causing the decreased expression of CD40, CD80, and CD86 and an inhibition of B cells activation. And co-incubation of this transfected B cells with autologous T cells could reduce the expression of T cell co-stimulatory molecules CD40L, CD28, and CD152, which are ligands of CD40, CD80, and CD86 on B cells. Then, we pretreated normal B cells with AKT inhibitor, then stimulated them with anti-IgM and anti-CD40 antibodies, and found that the phosphorylation level of AKT decreased while the protein level of P53 increased which led to the elevated expressional level of miR-1246 compared with anti-IgM- and anti-CD40-treated normal B cells. These implicated that activation of B cells could downregulate miR-1246 expression through the AKT-P53 signaling pathway, and decreased miR-1246 could aggravate B cells’ further activation by regulating EBF1, which means downregulation of miR-1246 in B cells is not just a consequence of increased lymphocyte activity, but also a potential cause of autoimmunity.

In the last part of the study, we found increased AKT phosphorylation while a downregulated P53 protein level in B cells from active SLE patients compared to healthy controls. P53 protein expression levels were negatively correlated with AKT phosphorylation levels and positively correlated with miR-1246 expression levels in B cells from active SLE patients. Furthermore, we treated active SLE B cells with AKT inhibitor and found that the phosphorylation level of AKT decreased and the protein level of P53 and miR-1246 expression increased. Yongxin Zhou *et al*. have reported that PI3K/AKT plays an important role in inhibition of spontaneous B apoptosis by downregulation of P53 [55]. Yu Zhang *et al*. investigate that miR-1246 is a new transcriptional target of the p53 by chromatin-associated immunoprecipitation assays, plasmid construction, and luciferase activity assay [27,28]. Thus, we conclude that since the p53 protein level is directly correlated with the miR-1246 expression, reduced miR-1246 levels in B cells of SLE patients may be due to inhibition of the P53 protein expression by AKT phosphorylation.

## Conclusions

The results from this study demonstrate the mechanistic relationship between B cell activation in SLE, decreased miR-1246 expression through AKT-P53 signaling pathway, and downstream effect on the expression of EBF1 leading to further activation of B cells. Therefore, therapies that turn the expression of affected miR-1246 genes back to normal could serve as a potential and effective method for treating SLE.

## Methods

### Samples

All subjects (active SLE patients: *n* = 30, inactive patients: *n* = 20, healthy controls: *n* = 20) recruited for this study were under a protocol approved by the Human Ethics Committee of Second Xiangya Hospital, Central South University, with an informed written consent. All SLE patients fulfilled the American College of Rheumatology (ACR) classification criteria for SLE [57]. Disease activity was assessed by the Systematic Lupus Activity Measure (SLAM) [58] and Systemic Lupus Erythematosus Disease Activity Index (SLEDAI) [59] at the time of blood collection. Patients who had active disease or inactive disease were selected based on the SLEDAI results (active: score above 5; inactive: score equal to or less than 4). Venous blood samples were collected from 30 active SLE patients (all female, age 32.7 ± 11.3 years), 20 inactive SLE patients (all female, age 30.3 ± 9.29 years) at the out-patient dermatology clinic and in-patient ward of the Department of Dermatology, the Second Xiangya Hospital, Central South University, China. Relevant patient information is listed in Additional file 1: Table S1. Twenty healthy controls with age and sex matched (all female, age 30.7 ± 8.6 years) were recruited from staff and graduate students at the Second Xiangya Hospital.

### Cell isolation, cultures, and transfection

A total of 60 ml of venous peripheral blood was withdrawn from each patient and control subject and preserved with heparin. B cells were isolated by positive selection using CD19 beads, according to protocols provided by the manufacturer (Miltenyi, BergischGladbach, Germany; purity was generally higher than 95%) and cultured in human B cell culture medium (Lonza, Walkersville, MD, USA) supplemented with 10% fetal bovine serum (FBS) and 1% penicillin/streptomycin. B Cells were transiently transfected with mimic/inhibitor control, has-mir-1246 mimic, or has-mir-1246 inhibitor (Ambion, USA) using Human B cell Nucleofector Kits and a nucleofector (Amaxa, USA) according to the manufacturer’s instructions. First, B cells were harvested and resuspended in 100-μl human B cell nucleofector solution, and then, the cell suspension was mixed with has-mir-1246 mimic/inhibitor or mimic/inhibitor control. The mix was electrotransfected using nucleofector program U-015 in the Amaxa nucleofector. The transfected cells were cultured in human B cell culture medium and harvested after 48 h.

### RNA isolation and real-time quantitative RT-PCR

Total RNA from B cells was isolated using TRIzol (Invitrogen Carlsbad, CA, USA). The relative abundance of gene expression was determined by real-time PCR using a Rotor-Gene 3000 (Corbett Research, NSW, Australia). cDNAs were synthesized from 1 μg of total RNA using the miScript Reverse Transcription Kit (Qiagen, Germany). DNA was synthesized from cDNA. Real-time PCR was performed using the Rotor-Gene 3000 Real-time PCR instrument (Corbett Research, Australia). All reactions were run in triplicate. Expression levels of target miRNAs were normalized to RUN6-2 and analyzed with Rotor-Gene Real-Time Analysis Software 6.0. MiR-1246, miR-126, miR-142-3p, miR-142-5p, and RUN6-2 primers were ordered from Qiagen. The information about the primers used for PCR are listed in Additional file 1: Table S2. ΔCt was calculated by subtracting the Ct values for RUN6-2 from the Ct value for the gene of interest. ΔΔCt was calculated by subtracting the control Ct from SLE Ct. The fold change of expression between control and SLE samples was calculated by the equation: 2^−ΔΔCt^.

### Plasmid construction and luciferase activity assay

A fragment sequence from the 3′-UTR of EBF1 containing a putative miRNA binding site was amplified by PCR from human B cell genomic DNA. The same procedure was used to generate reporter constructs with mutations in the 3′-UTR of the target gene. 3′-UTR sequences were inserted into pMIR-REPORT luciferase miRNA Expression Reporter Vector (Ambion) using Spe I and Hind III. The primers used for PCR are listed in Additional file 1: Table S2. Jurkat cells were cultured in RPMI 1640 with 10% FBS. Luciferase activity assays were performed as previously described [21] with 5 μg of firefly luciferase reporter vector containing either the wild-type or mutant oligonucleotides, 0.5 μl of mimic control, and has-mir-1246 mimic. Relative luciferase activity was normalized to renilla luciferase activity for each transfected well. Experiments were performed in triplicate in three independent trials.

### B cell activation

Purified B cells from healthy control were cultured in 6-well plates (1 × 10^6^/ml) and stimulated with anti-IgM (2 μg/ml) in the presence of anti-CD40 (0.1 μg/ml) and PBS (blank control), then incubated for 6 h at 37°C.

### AKT inhibition

Purified B cells from healthy control were cultured in 6-well plates (1 × 10^6^/ml) and stimulated with MK-2206 2HC (Selleck Chemicals, TX, USA), a specific AKT inhibitor or PBS (blank control); after being incubated for 6 h, anti-IgM (2 μg/ml) + anti-CD40 (0.1 μg/ml) were added into them, then incubated for 6 h at 37°C. Purified B cells from active SLE patients were cultured in 6-well plates (1 × 10^6^/ml) and stimulated with AKT inhibitor or PBS (blank control), then incubated for 6 h at 37°C.

### Western blot analysis

B cells were lysed in protein lysis buffer containing phosphatase inhibitor (Therm Pierce). Lysates were centrifuged for 15 min at 14,000 × *g* at 4°C, and protein concentration was determined by Bradford protein assay (Bio-Rad, CA, USA). Proteins were separated by SDS-PAGE using 8% polyacrylamide gels. Proteins were then transferred onto PVDF membranes (Millipore, MA, USA). Membranes were blocked with 5% non-fat dry milk in Tris-buffered saline containing 0.1% Tween-20 (TBST) buffer and immunoblotted with primary antibodies including anti-β-actin (Sigma, MA, USA), anti-EBF1 (Sigma, MA, USA), anti-AKT (Sigma, MA, USA), anti-pAKT (Sigma, MA, USA), and anti-P53 (Sigma, MA, USA). Band intensity was quantified using Quantity One software (Bio-Rad, CA, USA).

### Flow cytometric analysis

PE-Cy7-conjugated anti-human CD40, FITC-conjugated anti-human CD80, PerCP-Cy5.5-conjugated anti-human CD86, PE-Cy5-conjugated anti-human CD40L, APC-conjugated anti-human CD28, PE-conjugated anti-human CD152, APC-conjugated anti-human CD19, and FITC-conjugated anti-human CD4 were purchased from Becton Dickinson (USA). Data were acquired using a FACScalibur system (Becton Dickinson) and analyzed using CellQuest software (Becton Dickinson,).

### T-B cell co-cultures for conjugate and co-stimulation assays

Isolated normal CD4^+^T cells were cultured in RPMI 1640 medium with 10% FBS, 100 U/ml of penicillin G, and streptomycin. After stimulation with anti-IgM (2 μg/ml) in the presence of anti-CD40 (0.1 μg/ml), for 6 h, CD40, CD80, and CD86 were measured from partially stimulated B cells by flow cytometry with the cells stained at 4°C for anti-CD40, anti-CD80, anti-CD86, and anti-CD19 antibodies. Stimulated B cells were transfected with miR-1246 mimic or a mimic control, for 48 h, and then, treated B cells were co-cultured with autologous CD4^+^T cells at a ratio of 4:1 in 96-well round-bottomed plates for 24 h; CD40L, CD28, and CD152 were then measured by flow cytometry with the cells stained at 4°C for anti-CD40L, anti-CD28, anti-CD152, and anti-CD4 antibodies.

### Statistical analysis

All statistical analyses were conducted by SPSS 16.0 software. Results were expressed as mean ± SD. Variables were compared by Student’s *t*-test (data from different transfections were compared by paired *t*-test, others by two-group *t*-test). Correlations were determined using Pearson’s correlation coefficient. *P* < 0.05 was considered significant.

## Additional files

Additional file 1:
**Supplementary tables on patient demographics and medications and primers used for PCR.** **Table S1.** Patient demographics and medications. **Table S2**. Primers for PCR. Additional file 2:
**Supplementary figures on the actual flow cytometry profiles.** **Figure S1.** represents for the actual flow cytometry profiles of Figure 4A, B. **Figure S2.** represents for the actual flow cytometry profiles of Figure 4C, D. **Figure S3.** represents for the actual flow cytometry profiles of Figure 5D, E. **Figure S4.** represents for the actual flow cytometry profiles of Figure 6D, E. **Figure S5.** represents for the actual flow cytometry profiles of Figure 6F, G.

## Abbreviations

AKT: also known as protein kinase B
CD40L: CD40 ligand
EBF1: early B cell factor 1
P53: also known as protein 53 or tumor protein 53
pAKT: phosphorylation of AKT
PBS: phosphate buffer saline
PI3K: phosphoinositide 3-kinase
SD: standard deviation
SLE: Systemic lupus erythematosus
