# Supplementary material for: The role of microRNA-1246 in the regulation of B cell activation and the pathogenesis of systemic lupus erythematosus
Source: Clin Epigenetics. 2015 Mar 14;7(1):24. doi: 10.1186/s13148-015-0063-7 (PMC4364674; doi:10.1186/s13148-015-0063-7)
Supplement: Additional file 1: — Supplementary tables on patient demographics and medications and primers used for PCR. Table S1. Patient demographics and medications. Table S2. Primers for PCR. [file 13148_2015_63_MOESM1_ESM.doc]

**Table S1** Patient demographics and medications

| Patient | Age | Sex | SLEDAI | Medications |
| --- | --- | --- | --- | --- |
| 1 | 36 | F | 18 | None |
| 2 | 48 | F | 14 | Pred 10 mg/d |
| 3 | 25 | F | 10 | Pred 5 mg/d |
| 4 | 15 | F | 8 | None |
| 5 | 16 | F | 16 | TG 30 mg/d |
| 6 | 48 | F | 16 | None |
| 7 | 47 | F | 18 | Pred 5 mg/d |
| 8 | 37 | F | 12 | None |
| 9 | 41 | F | 24 | Pred 15 mg/d |
| 10 | 16 | F | 20 | TG 30 mg/d, Pred 10 mg/d |
| 11 | 40 | F | 26 | Pred 10 mg/d |
| 12 | 27 | F | 18 | Pred 20 mg/d ,HCQ 0.2 g/d |
| 13 | 27 | F | 16 | None |
| 14 | 21 | F | 12 | None |
| 15 | 40 | F | 15 | Pred 15 mg/d |
| 16 | 46 | F | 14 | Pred 20 mg/d |
| 17 | 36 | F | 14 | TG 30 mg/d, HCQ 0.2 g/d |
| 18 | 42 | F | 10 | None |
| 19 | 22 | F | 20 | Pred 20 mg/d ,HCQ 0.2 g/d |
| 20 | 42 | F | 20 | Pred 15 mg/d,HCQ 0.2 g/d |
| 21 | 28 | F | 20 | None |
| 22 | 48 | F | 24 | None |
| 23 | 44 | F | 16 | Pred 10 mg/d |
| 24 | 25 | F | 16 | None |
| 25 | 16 | F | 22 | Pred 15 mg/d |
| 26 | 27 | F | 26 | Pred 15 mg/d |
| 27 | 20 | F | 16 | None |
| 28 | 28 | F | 20 | Pred 10 mg/d |
| 29 | 26 | F | 32 | Pred 15 mg/d |
| 30 | 48 | F | 16 | TG 30 mg/d, Pred 10 mg/d |
| 31 | 29 | F | 2 | None |
| 32 | 22 | F | 2 | None |
| 33 | 16 | F | 4 | None |
| 34 | 27 | F | 2 | Pred 5 mg/d |
| 35 | 38 | F | 4 | Pred 5 mg/d |
| 36 | 20 | F | 2 | None |
| 37 | 45 | F | 2 | None |
| 38 | 35 | F | 4 | Pred 5 mg/d |
| 39 | 43 | F | 2 | Pred 5 mg/d |
| 40 | 25 | F | 2 | None |
| 41 | 47 | F | 4 | None |
| 42 | 24 | F | 2 | Pred 5 mg/d |
| 43 | 37 | F | 4 | Pred 5 mg/d |
| 44 | 26 | F | 0 | None |
| 45 | 39 | F | 0 | None |
| 46 | 24 | F | 2 | Pred 5 mg/d |
| 47 | 31 | F | 0 | None |
| 48 | 17 | F | 0 | None |
| 49 | 24 | F | 2 | None |
| 50 | 37 | F | 4 | Pred 5 mg/d |

F, female; M, male; SLEDAI, SLE Disease Activity Index; Pred, prednisone; TG, tripterygium glycoside; HCQ, hydroxychloroquine

**Table S2** Primers for PCR

| **Primer name** | **Primer sequence (5’-3’)** |
| --- | --- |
| EBF1 WT-luciferase (F) | TTactagtTCACAAAAAGATTTTACTGCGTATT |
| EBF1 WT-luciferase (R) | ATaagcttTTATATGGATATTTTACAAAATCCC |
| EBF1 Mut-luciferase (F) | TTactagtTCACAAAAAGATTTTACTGCGTATT |
| EBF1 Mut-luciferase (R) | ATaagcttTTATATCCGTATTTTACAAAATCCC |
| RUN6-2 Forward | CTCGCTTCGGCAGCACA |
| RUN6-2 Reverse | AACGCTTCACGAATTTGCGT |

Lower case letters represent restriction enzyme recognition sites.
