# Supplementary figures and images for: The role of microRNA-1246 in the regulation of B cell activation and the pathogenesis of systemic lupus erythematosus
Source: Clin Epigenetics. 2015 Mar 14;7(1):24. doi: 10.1186/s13148-015-0063-7 (PMC4364674; doi:10.1186/s13148-015-0063-7)

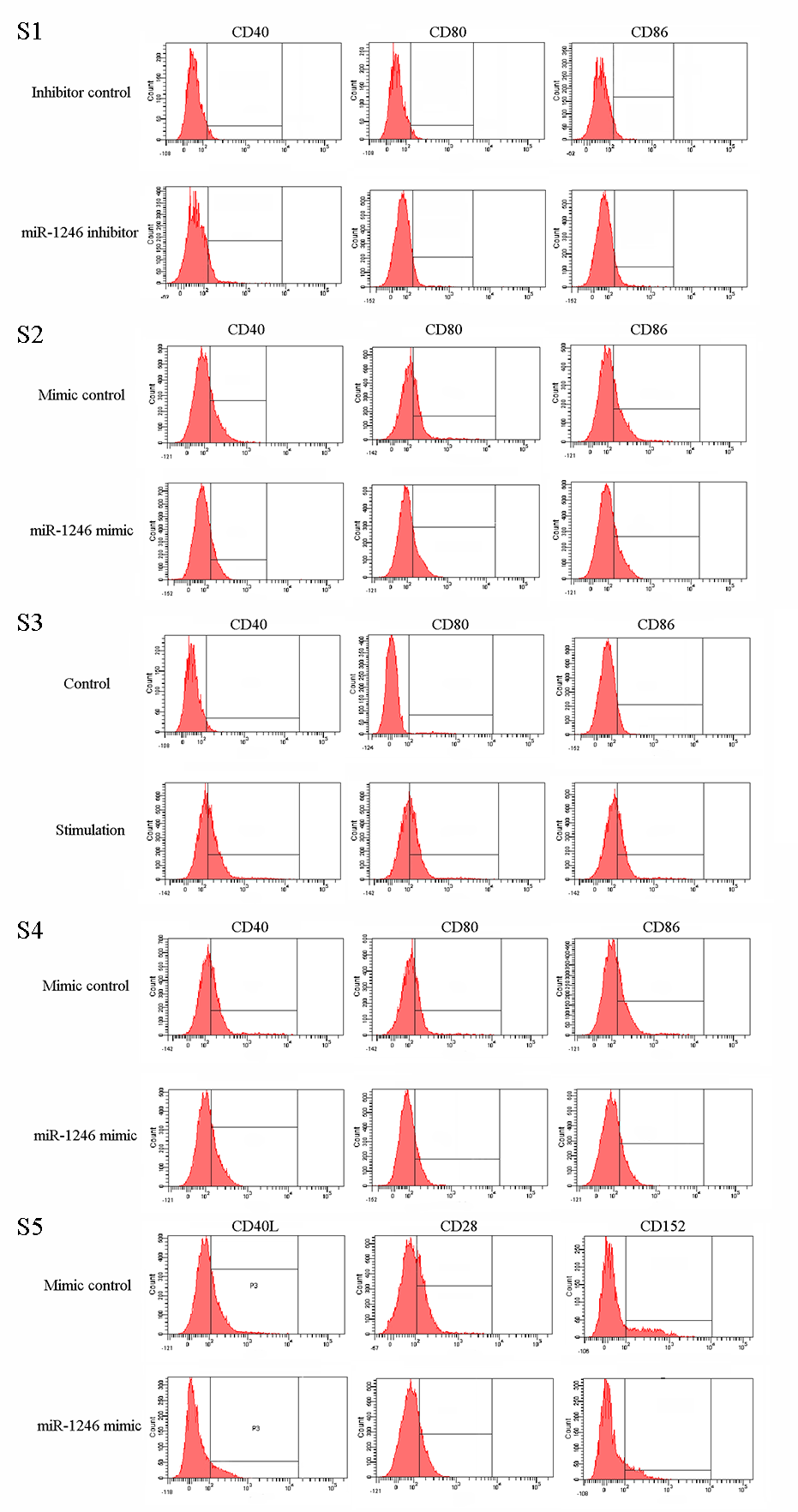

Supplement: Additional file 2: — Supplementary figures on the actual flow cytometry profiles. Figure S1. represents for the actual flow cytometry profiles of Figure 4A, B. Figure S2. represents for the actual flow cytometry profiles of Figure 4C, D. Figure S3. represents for the actual flow cytometry profiles of Figure 5D, E. Figure S4. represents for the actual flow cytometry profiles of Figure 6D, E. Figure S5. represents for the actual flow cytometry profiles of Figure 6F, G. [file 13148_2015_63_MOESM2_ESM.tiff]
